# Interaction of a Partially Disordered Antisigma Factor with Its Partner, the Signaling Domain of the TonB-Dependent Transporter HasR

**DOI:** 10.1371/journal.pone.0089502

**Published:** 2014-04-11

**Authors:** Idir Malki, Catherine Simenel, Halina Wojtowicz, Gisele Cardoso de Amorim, Ada Prochnicka-Chalufour, Sylviane Hoos, Bertrand Raynal, Patrick England, Alain Chaffotte, Muriel Delepierre, Philippe Delepelaire, Nadia Izadi-Pruneyre

**Affiliations:** 1 Institut Pasteur, Unité de RMN des Biomolécules, Département de Biologie Structurale et Chimie, Paris, France; 2 CNRS, UMR 3528, Paris, France; 3 Université Pierre et Marie Curie, Cellule Pasteur UPMC, Paris, France; 4 Institut Pasteur, Plate-forme de Biophysique des Macromolécules et de leurs Interactions, Département de Biologie Structurale et Chimie, Paris, France; 5 Institut de Biologie Physico-Chimique, CNRS Université Paris-Diderot UMR 7099, Paris, France; Centre National de la Recherche Scientifique, Aix-Marseille Université, France

## Abstract

Bacteria use diverse signaling pathways to control gene expression in response to external stimuli. In Gram-negative bacteria, the binding of a nutrient is sensed by an outer membrane transporter. This signal is then transmitted to an antisigma factor and subsequently to the cytoplasm where an ECF sigma factor induces expression of genes related to the acquisition of this nutrient. The molecular interactions involved in this transmembrane signaling are poorly understood and structural data on this family of antisigma factor are rare. Here, we present the first structural study of the periplasmic domain of an antisigma factor and its interaction with the transporter. The study concerns the signaling in the heme acquisition system (Has) of *Serratia marcescens*. Our data support unprecedented partially disordered periplasmic domain of an anti-sigma factor HasS in contact with a membrane-mimicking environment. We solved the 3D structure of the signaling domain of HasR transporter and identified the residues at the HasS−HasR interface. Their conservation in several bacteria suggests wider significance of the proposed model for the understanding of bacterial transmembrane signaling.

## Introduction

Bacteria need external sensors and signaling processes that enable them to detect environmental changes (light, oxidative stress, availability of some nutrients, etc) and to respond by differential gene expression. These specific genes are regulated by a class of sigma factors named ECF sigma factors (for ExtraCytoplasmic Function) [1].

The ECF sigma factors, a subset of the sigma 70 superfamily which encompasses almost 60% of all bacterial sigma factors, control gene expression at the level of transcription initiation by recruiting RNA polymerase. Their capacity to bind the RNA polymerase is generally regulated by cognate antisigma factors that are in most cases transmembrane proteins. In the absence of the signal, the antisigma factor binds to the ECF sigma factor, thereby keeping it inactive. Upon receiving the stimulus, the antisigma factor becomes inactive, either by degradation through a cascade of regulated proteolytic steps, or through conformational changes [2], [3]. Both mechanisms result in release and consequent activation of the ECF sigma factor, which can then induce gene expression of its target promoters [1].

One group of genes regulated by this signaling pathway is involved in iron acquisition by Gram-negative bacteria. In these systems, binding of the substrate (either a siderophore, a hemoprotein or heme) by an outer membrane transporter stimulates an antisigma factor which transduces a signal to a cytoplasmic ECF sigma factor. We have studied one of these systems named Has (for Heme acquisition system), developed by several Gram-negative bacteria to acquire heme as an iron source. Here, we focus on the Has system of *Serratia marcescens*, the first identified and most deeply studied [4].

The central protein of the Has system, the outer membrane transporter HasR functions in synergy with an extracellular heme carrier protein, the hemophore HasA. HasA either binds free heme or captures it from host hemoproteins and then delivers it to HasR. Heme is then internalized whilst the hemophore is released into the extracellular medium. Energy, brought by HasB an inner membrane TonB like protein, is required for the activity of HasR [5].

Consistent with most of the bacterial iron/heme acquisition systems, the expression of the *has* operon encoding the Has system proteins, is negatively regulated by the intracellular concentration of iron *via* Fur (ferric uptake regulator). In some systems, including Has, Fec, Pup, Pvd, Bhu, an additional level of regulation is fulfilled by an antisigma/ECF sigma factor couple as described above [6]. In the Has system, the stimulus is the concomitant presence of heme and HasA on the extracellular side of the outer membrane transporter HasR [7]. The binding of these two ligands triggers a signal that is transmitted by the periplasmic domain of HasR to the antisigma factor, HasS, through which it finally reaches the cytoplasm where the ECF sigma factor, HasI, induces the expression of the *has* operon. In the absence of extracellular substrates, the activity of HasI is inhibited, most probably due to its sequestration by HasS. The presence of the HasB/TonB complex is required for this signaling pathway [8].

The periplasmic domain of HasR located at its N-terminal extremity has not been seen in the crystal structure of the receptor suggesting that this region is either disordered or flexible with respect to the remainder of the protein [9]. This part of HasR is composed of the signaling domain and a linker of 21 residues containing the TonB/HasB box, a critical region for the interaction with the energy transducer protein [10]. The signaling domain of HasR, like other signaling domains of TonB dependent transporters, is only required for its regulating activity and is not involved in the transport function [11].

The antisigma factor HasS plays a key role in this signaling pathway. As other antisigma factors involved in heme/iron uptake regulation, HasS possesses a putative transmembrane helix (residue 85 to 101), which anchors it into the inner membrane. On each side of this transmembrane helix, are located the N-terminal cytoplasmic and the C-terminal periplasmic domains, comprising respectively 84 and 217 residues (Figure S1 in File S1). Using bacterial two-hybrid system and mutagenesis studies on HasS homologs (FecR, RhuR, FpvR, etc) sharing about 40% of sequence identity, it has been shown that the N-terminal domain is in charge of the regulation of the ECF sigma factor activity whereas the C-terminal domain receives the stimulus from the signaling domain of the transporter [12], [13]. Likewise only the last 80 residues of FecR have been shown to be compulsory for the interaction with the signaling domain of the transporter FecA [12].

The structural organization of the antisigma factors through the inner membrane enables communication between cell compartments. However it renders the structural study of these proteins difficult. As a result, there are only sparse structural data available on these proteins, concerning exclusively their cytoplasmic domain. The mechanism by which the antisigma factors sense the signal from the extracellular medium is not understood and the structure of their domain responsible for stimulus detection remains unknown.

Here, we present the first structural study of an antisigma factor periplasmic domain, HasS_CTD_. We show that this domain is partially disordered, needs to be in contact with a membrane mimicking environment and that its structural features are compatible with its activity. We also solved by NMR the 3D structure of its partner, the periplasmic domain of HasR. The study of the interaction between these two protein domains allows to propose a model of the propagation of the external signal *via* the outer membrane transporter.

## Materials and Methods

### Protein preparation

The DNA fragment encoding the last 78 residues of HasS (HasS_CTD_) was cloned into a pETM-11 expression vector (*Novagen*) using standard cloning protocols. This vector directs expression of an N-terminal His6 tag followed by a TEV protease cleavage site, three additional residues (GAM) and HasS_CTD_. The obtained expression vector was transformed into *E. coli* BL21(DE3)pLysS cells. For ^13^C, ^15^N-labeled protein samples, cells were grown at 37°C in M9 medium containing 0.1% ^15^NH_4_Cl and 0.4% ^13^C-glucose, as the only nitrogen and carbon sources, respectively. Protein overexpression was induced with 1 mM IPTG (isopropyl β-D-thiogalactopyranoside) at OD_600_ around 0.8. Cells were harvested after 4 h from induction, resuspended, lysed by sonication and centrifuged (20000×g for 40 min) to sediment the inclusion bodies. The pellet was washed and centrifuged four times with 3% triton solution containing 1.5 M NaCl. Finally the washed pellet was solubilized in 0.1 M Tris-HCl pH 8 buffer containing 8 M urea. The obtained supernatant was diluted with 50 mM sodium phosphate buffer pH 7 to the final urea concentration of 3 M and incubated overnight with TEV protease. After TEV cleavage, urea concentration was decreased to 0.7 M by dilution in the renaturation buffer (50 mM sodium buffer, pH 7, 50 mM NaCl, 0.05% Zwittergent 3-14) and finally removed by dialysis against the renaturation buffer. The protein concentration at this step was around 10 µg/ml. Proper refolding of the protein could be only obtained in presence of a detergent. Several detergents (Chaps, Tween, Triton ×100, Zwittergent) were tested, the most efficient being Zwittergent 3-14 (Zw) at 0.05% (w/v). At this concentration, the micelles of Zw are present in the buffer. HasS_CTD_ was finally purified using a nickel affinity chromatography column [*GE Healthcare Life Science*) followed by a size exclusion column (Sephacryl S-100 HP 16/60, *GE Healthcare Life Science*) in the renaturation buffer. All the purification steps were performed at 4°C in the presence of a protease inhibitor cocktail (*Roche*). The above protocol yielded about 20 mg of HasS_CTD_ (>95% pure) per liter of culture. HasS_CTD_ concentration was estimated from its absorbance at 280 nm assuming a calculated ε280 of 13980 M^−1^.cm^−1^.

Overexpression and purification of the periplasmic domain of HasR, composed of the signaling domain and a stretch of 21 residues, were carried out as described previously [14]. The same expression and purification protocols were used to produce the signaling domain alone with only two additional N-terminal residues (SM). The signaling domain was only used for probing protein-protein interaction after having verified, by superimposing ^15^N-^1^H HSQC spectra of both constructs, that the structure of this domain is conserved either with or without the stretch of 21 residues. The concentrations of these domains were determined by their amino acid analysis after 6 N HCl hydrolysis for 24 h at 110°C.

### NMR spectroscopy

All NMR experiments were recorded on a Varian spectrometer operating at a proton frequency of 600 MHz and equipped with a cryogenically cooled triple resonance 1H{13C/15N} PFG probe. The pulse sequences of experiments were taken as implemented from the VnmrJ Biopack (Agilent Technologies, http://www.chem.agilent.com/). All NMR spectra were processed using NMRPipe [15] and analyzed by CcpNmr Analysis [16]. The HasS_CTD_ samples concentrations used for NMR experiments ranged from 30 to 250 µM (depending on the experiment) in 50 mM sodium phosphate buffer, pH 7, 50 mM NaCl, 0.05% Zw, 15% D_2_O.

HasS_CTD_ backbone resonance assignments were done using the following triple resonance experiments: HNCO, CBCA(CO)NH and HNCACB. In order to get optimal signal with narrower linewidth, experiments were carried out at 45°C and pH 5.8. Chemical shift index (CSI) values were determined using the method of Wishart and Sykes [17].

The backbone ^15^N dynamics experiments of the periplasmic domain of HasR and HasS_CTD_ were performed at 20°C. A series of relaxation delay times between 20 and 1500 ms was used in the T1 experiments, while delay times ranging from 10 to 170 ms were used in the T2 measurements. R1 and R2 relaxation rates were estimated by fitting the T1 and T2 peak intensities to a single exponential decay. The error in the relaxation rate was obtained from the error of the fit of the data. ^1^H-^15^N steady-state NOE values were determined as the ratio of the peak intensities in the spectra recorded with and without pre-saturation of ^1^H. NOE errors were estimated from the noise standard deviation. The HasS_CTD_ sample used for relaxation experiments was 250 µM in 50 mM sodium phosphate buffer, pH 7, 50 mM NaCl, 0.05% Zw, 15% D_2_O. The concentration of the periplasmic domain of HasR sample was 400 µM in 50 mM sodium phosphate buffer, pH 7, 50 mM NaCl, 15% D_2_O. R1, R2 and NOE of the periplasmic domain of HasR were analysed in terms of internal motions using the program TENSOR [18].

Deuterium/proton exchange experiments were carried out with a sample of 100 µM of ^15^N labeled HasS_CTD_ in 50 mM sodium phosphate buffer, pH 7, 50 mM NaCl, 0.05% Zw in presence of either 15% or 100% D_2_O (after lyophilization). A control experiment with a HasS_CTD_ sample lyophilized and resuspended in H_2_O was recorded to verify that the lyophilization did not alter the protein.

### Circular Dichroism spectroscopy (CD)

All CD measurements were acquired using an Aviv 215 spectropolarimeter. Far-UV (195–260 nm) spectra were recorded at 20°C on a 50 µM HasS_CTD_ sample in 50 mM sodium phosphate buffer, pH 7, 50 mM NaCl, 0.05% Zw using a 0.02 cm path-length cylindrical cell. Ellipticity was measured every 0.5 nm with an averaging time of 2 s. Spectrum of the protein sample resulted from averaging three successive scans followed by subtraction of the baseline spectrum of buffer recorded under the same conditions. Thermal unfolding of HasS_CTD_ was executed by heating of a 5 µM protein solution in the same buffer as above from 20 and 90°C in a 0.1 cm rectangular cell, using a temperature increase rate of 1°C/min. Ellipticity change was recorded every 5°C using the same instrument settings as described above. The quantitative decomposition of the far-UV CD spectrum of HasS_CTD_ was done using CONTIN program [19].

### Analytical ultracentrifugation

Sedimentation velocity experiments were performed at 20°C in a Beckman Coulter XL-I analytical ultracentrifuge equipped with double UV and Rayleigh interference detection. The HasS_CTD_ samples (100 µL at 86 µM and 300 µL at 14 µM) were loaded in 3 and 12 mm thick double sector epoxy centerpieces, respectively whereas the solvent containing detergent was loaded in a 12 mm Centerpiece. Solvent without detergent was loaded as reference in the double sector centerpieces. Samples were spun at 42 000 rpm using an An60Ti rotor. The partial specific volume of HasS (0.739 mL/g) was estimated from its amino acid sequence using the software Sednterp (v 1.09) and the partial specific volume of detergent was taken as 0.971. Density and viscosity of the buffer, which are required for the analysis, were calculated with the same software. Sedimentation profiles were acquired overnight every 3 min using absorbance at 280 nm and interference optics. Velocity experiments data were analyzed using the software SEDFIT 13.0b (available on www.analyticalultracentrifugation.com) with the continuous distribution c(S) model [20].

The amount of each partner in each peak from c(s) and consequently the stoichiometry of the protein/detergent complex were calculated as previously published [21]. Briefly, in each peak the signal of absorbance or interference (fringe shift) obey the Beer Lamber law. It is related to concentration, optical path length, molar extinction coefficient for absorbance measurement and molar refractive increment (∂n/∂c) as well as laser wavelength (λ = 675 nm) for interference measurement. At wavelength of 280 nm extinction coefficient of the HasS_CTD_ was 13980 M^−1^.cm^−1^, and zero for the detergent, whereas the refractive increment was 0.185 mL/g for HasS_CTD_ and 0.156 mL/g for the detergent.

### Interaction of HasS_CTD_ with the detergent micelles

The broadening of signals induced by the paramagnetic effect of Mn^2+^ was analyzed by measuring the peak intensity of the protein before and after addition of MnCl_2_. ^15^N-^1^H HSQC spectra of a sample of 100 µM HasS_CTD_ were recorded before and after addition of 7 µL of MnCl_2_ at 50 mM (final concentration of 2 mM). The HasS_CTD_ sample was in 50 mM Tris, pH 7, 50 mM NaCl, 0.05% Zw because of the low solubility of Mn2+ in phosphate buffer.

### Structure calculation of the periplasmic domain of HasR-

NMR experimental data were handled under CcpNmr Analysis [16]. Almost all backbone and side chains resonances were assigned, as described in our previous work [14] and deposited to the BMRB (entry code: 18201). The structure of HasR periplasmic domain was calculated using NOE-derived distance constraints, dihedral angles and hydrogen bonds. Distance constraints were obtained from 3D ^13^C and ^15^N NOESY-HSQC experiments with mixing times of 100 and 80 ms, respectively. Dihedral angle restraints were determined from backbone chemical shifts using Analysis DANGLE program. Hydrogen bond donors were obtained from a series of ^1^H-^15^N HSQC experiments of lyophilized protein dissolved in D_2_O and monitored for 36 hours. The corresponding hydrogen bond acceptors were identified based on the NOE pattern from the 3D HSQC-NOESY experiments.

Structure calculation was combined with automatic NOE cross-peak assignment using ARIA version 2.2 and CNS version 1.2 [22], [23]. Several cycles of ARIA were performed using standard protocols with spin diffusion corrections. After each cycle, rejected restraints, assignments and violations were analyzed. Finally, from 400 conformers calculated with ARIA/CNS, 200 were refined in water, and 20, with the lowest restraint energy values were used for statistical analysis. The structures were visualized and analysed with MOLMOL and PYMOL (DeLano, W.L., Copyright 2009–2010 Schrödinger LLC). Their quality was assessed using PROCHECK and WHATCHECK [24], [25].

### Protein-protein interaction

HasS_CTD_ interaction with the HasR signaling domain was studied using fluorescence spectroscopy at 20°C. All the spectra were recorded at Perkin Elmer LS50B Spectrofluorimeter. Samples were excited at 280 and 297 nm with an excitation bandwidth of 2.5 nm (emission bandwidth of 2.5 nm) and the emission spectra were recorded from 310 to 450 nm with a scan speed of 60 nm/min. For protein-protein interaction experiments aliquots of a 1 mM solution of the HasR signaling domain in 20 mM phosphate buffer, pH 7 were added to 150 µL solution of 86 µM HasS_CTD_ in 50 mM sodium phosphate buffer, pH 7, 50 mM NaCl, 0.05% Zw. After addition of each aliquot, the sample was mixed and incubated for 5 min before recording the fluorescence emission spectrum. In control experiments, addition of HasR signaling domain was replaced by addition of the equivalent volume of either buffer or a solution of Ribonuclease A (*Sigma*) at 1.1 mM. All spectra were corrected for the internal filter effect and presented results are average of three independent measurements.

^1^H-^15^N HSQC spectra were recorded to probe the interaction between HasS_CTD_ and the signaling domain of HasR when only one of them was alternatively ^15^N labeled. The intensity of peaks of labeled protein was measured before and after addition of its unlabeled partner. It is noteworthy that we tried to remain at the highest possible concentration of proteins to favor formation of the complex. However, the protein concentrations used in this study were limited by the HasS_CTD_ maximum concentration we reached, i.e. 250 µM. In order to avoid the dilution of HasS_CTD_ and to keep it at its highest concentration, we added the signaling domain, either labeled or unlabeled, in a lyophilized form. When HasS_CTD_ was ^15^N labeled, a sample of 250 µM (in 50 mM sodium phosphate buffer, pH 7, 50 mM NaCl, 0.05% Zw and 15% D_2_O) was mixed with a lyophilized form of the signaling domain of HasR in 20 mM sodium phosphate buffer pH 7. Three NMR samples containing 250∶200 µM, 250∶400 µM, and 250∶800 µM of HasS_CTD_/signaling domain were analyzed. A control spectrum of HasS_CTD_ in the final buffer (70 mM sodium phosphate buffer, pH 7, 50 mM NaCl, 0.05% Zw and 15% D_2_O) was also recorded. To probe the interaction on the signaling domain side, a lyophilized form of this protein at 20 mM sodium phosphate buffer, pH 7 was added to a sample containing either unlabeled 250 µM HasS_CTD_ (50 mM sodium phosphate buffer, pH 7, 50 mM NaCl, 0.05% Zw and 15% D_2_O), or only the buffer, as the control. The final concentration of ^15^N signaling domain in these conditions was 100 µM. All titration experiments were performed at 20°C. Volume of the samples before the lyophilization and in the NMR tube was the same to avoid changes of buffer and salt concentration. A control experiment with HasS_CTD_ and the periplasmic domain of HasR (signaling domain plus the linker containing the HasB/TonB box) has been carried out to see whether the presence of the linker has an impact on this protein-protein interaction. For this test, the concentrations of HasS_CTD_ and the periplasmic domain of HasR were respectively 250 and 400 µM.

### Activity of HasS_CTD_ in S. marcescens

Plasmid pBADHasS_CTD_, encoding the HasS_CTD_ cloned downstream of the HasR signal sequence was constructed by Proteogenix (*Schiltigheim, France). S. marcescens* SM365 was respectively transformed with plasmids pBAD24 and pBADHasSCTD, to yield 30°C in M9 minimal medium in the presence of 1 mg/ml ampicillin with either 0.4% glucose and ferric citrate (30 µM), or 0.4% glycerol, or 0.4% glycerol and 0.4% arabinose. After one hour of growth at 30°C, dipyridyl (0.25 mM final) was added to the glycerol samples. Hemin-BSA was added at a concentration of 200 nM, 90 minutes after the addition of Dipyridyl. After a further 90 minutes, the OD600 nm of the different samples was measured and 10 ml of cell suspension were precipitated with TCA (20% final concentration). The equivalent of 0.15 OD_600nm_ was analyzed by gel electrophoresis and Western blot using either anti-HasR, or anti-HasA antibodies as previously described [26], [27].

## Results

### Expression of HasS_CTD_ in inclusion bodies and its refolding

HasS_CTD_ containing the last 78 residues of HasS, is the equivalent of the shortest domain of FecR defined as the domain of interaction with the transporter [12], [28]. HasS_CTD_ was found exclusively in inclusion bodies and could not be detected in any significant amount in the soluble extract. Although we used several approaches (variation of domain boundaries, modification of the expression strategy, etc), the recombinant protein was always found to be insoluble. After testing several refolding strategies (dialysis, dilution, typical low molecular weight additives and on-column refolding), the refolding method summarized below was conclusive.

After solubilisation of the inclusion bodies in 8 M urea, HasS_CTD_ renaturation was initiated by the dilution of urea to 3 M. This step of dilution to an intermediate concentration of denaturant was essential to limit the aggregation of the protein during the refolding process. The rest of the denaturant was then removed by a second step of dilution to 0.7 M urea followed by dialysis. At this step, the only way to limit protein aggregation was to add the detergent. At the last step of the purification process using a size exclusion column, an isolated elution peak containing pure HasS_CTD_ was obtained (Figure S2 in File S1).

### Structural features of HasS_CTD_

**Figure 1:**
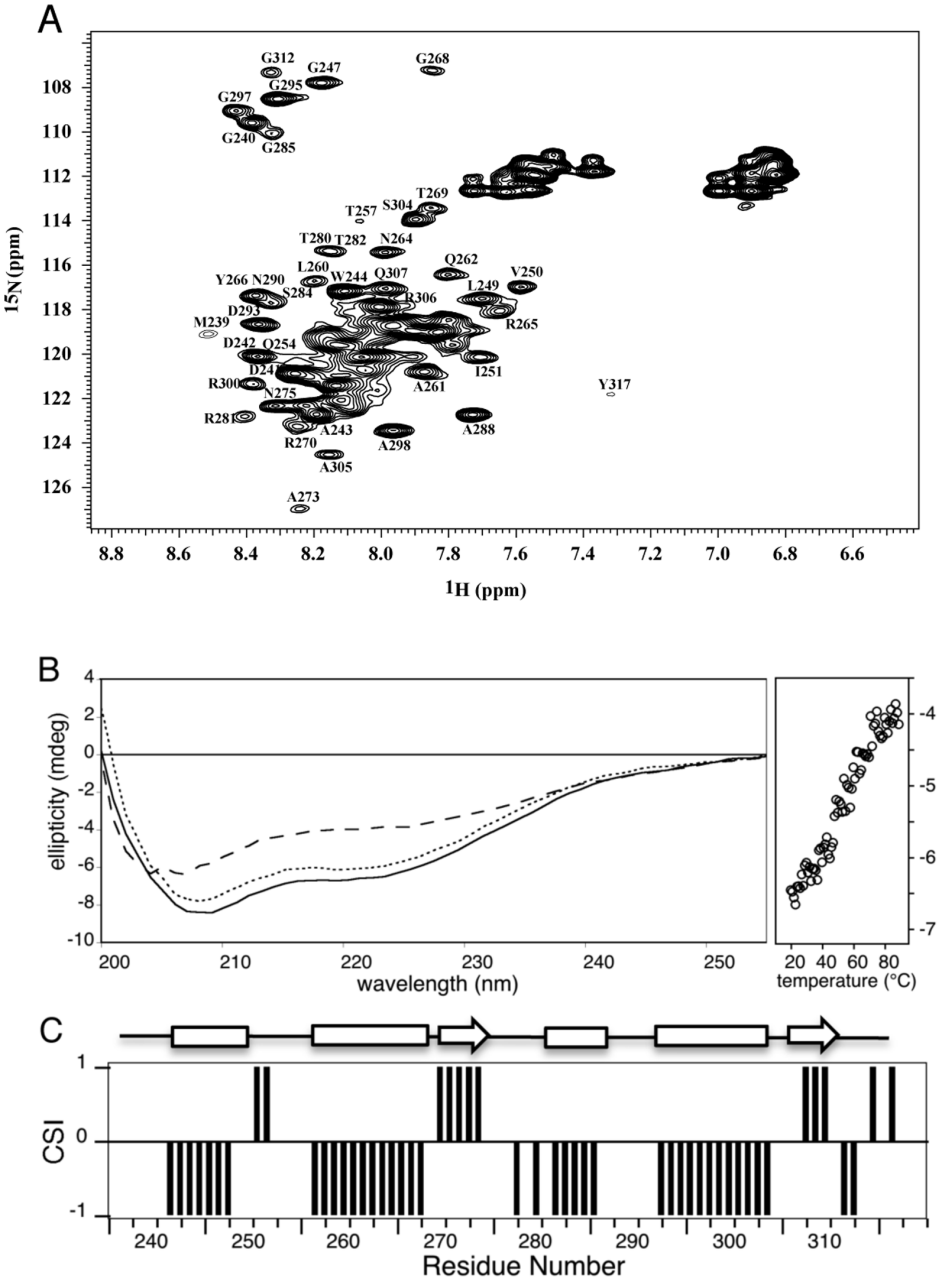
Structural characterization of HasS_CTD_. (A) ^15^N-^1^H HSQC spectrum of a sample of HasS_CTD_ at 250 µM in 50 mM sodium phosphate buffer, pH7, 50 mM NaCl, 0,05% Zw. Backbone resonance assignments of isolated peaks are indicated in one-letter amino acid code. (B) Far-UV CD spectra of the HasS_CTD_ protein at 20°C (solid line), at 90°C after thermally induced unfolding (dashed line) and the refolded protein at 20°C (dotted line). Loss of the HasS_CTD_ secondary structure (change of the ellipticity at 222 nm) with increasing temperature is shown on the right panel. (C) Chemical shift index (*CSI*) plot of HasS_CTD_. The values of *CSI* for β-strand, α-helix and random coil are +1, −1 and 0, respectively. Secondary structure elements, displayed on the top, were obtained from a consensus of *CSI* and DANGLE.

The first structural information about HasS_CTD_ was obtained by analysis of its ^15^N-^1^H HSQC spectrum (Figure 1A). The NMR resonances are broad, indicating either a high molecular weight due to oligomerization or the presence of multiple conformations. Moreover, the ^1^H resonances are not well dispersed (“crowded” between 7.5–8.6 ppm) suggesting that at least a significant portion of the protein is disordered.

The oligomeric state of HasS_CTD_ was investigated by sedimentation velocity analytical ultracentrifugation at the same range of protein concentration as that used for NMR. Analysis of the data showed that HasS_CTD_ is in a monomeric form sedimenting at 1.0±0.1 S. From this value, a hydrodynamic radius of 25 Å was calculated. The HasS_CTD_ frictional ratio of 1.5 suggests that its shape is not globular and that it may adopt an elongated form.

Further structural information was obtained by CD and NMR spectroscopy. The quantitative decomposition of the far-UV CD spectrum of HasS_CTD_ resulted in 33% helical and 11% of extended structure contents (Figure 1B). In order to obtain residue specific structural information, NMR assignments were carried out. Because of the broadening of NMR signals, only the set of three-dimensional heteronuclear experiments for backbone assignments could be operated. Almost all ^1^H, ^15^N, ^13^Cα, ^13^Cβ, ^13^Co resonances were assigned, except those of L256, T314 and L316 (Table S1 in File S1).

The residue-specific secondary structure propensities of HasS_CTD_ were then determined using the CSI and DANGLE methods (Figure 1C and S3). Their analysis reveals existence of four helices (H1: D241-W248; H2: T257-R267; H3: T282-V286; H4: D293-S304) and two short β-strands (β1: R270-V274; β2: L308-L310).

To gain insight into the tertiary structure of HasS_CTD_, hydrogen-deuterium exchange experiments were carried out using NMR. After one hour, all backbone amide resonances disappeared from the spectrum (data not shown), indicating that they were not involved in stable hydrogen bonds and that the whole protein was accessible to the bulk solvent. In addition, this apparent lack of tertiary structure was confirmed by monitoring thermal denaturation of HasS_CTD_ by far-UV CD. No cooperative transition typical of globular proteins but only gradual loss of the helical structures was noticed (Figure 1B). All these results and observations demonstrate that HasS_CTD_ is an elongated monomer, displaying distinct secondary structure elements but lacking stable tertiary structure.

### Backbone dynamics of HasS_CTD_

**Figure 2:**
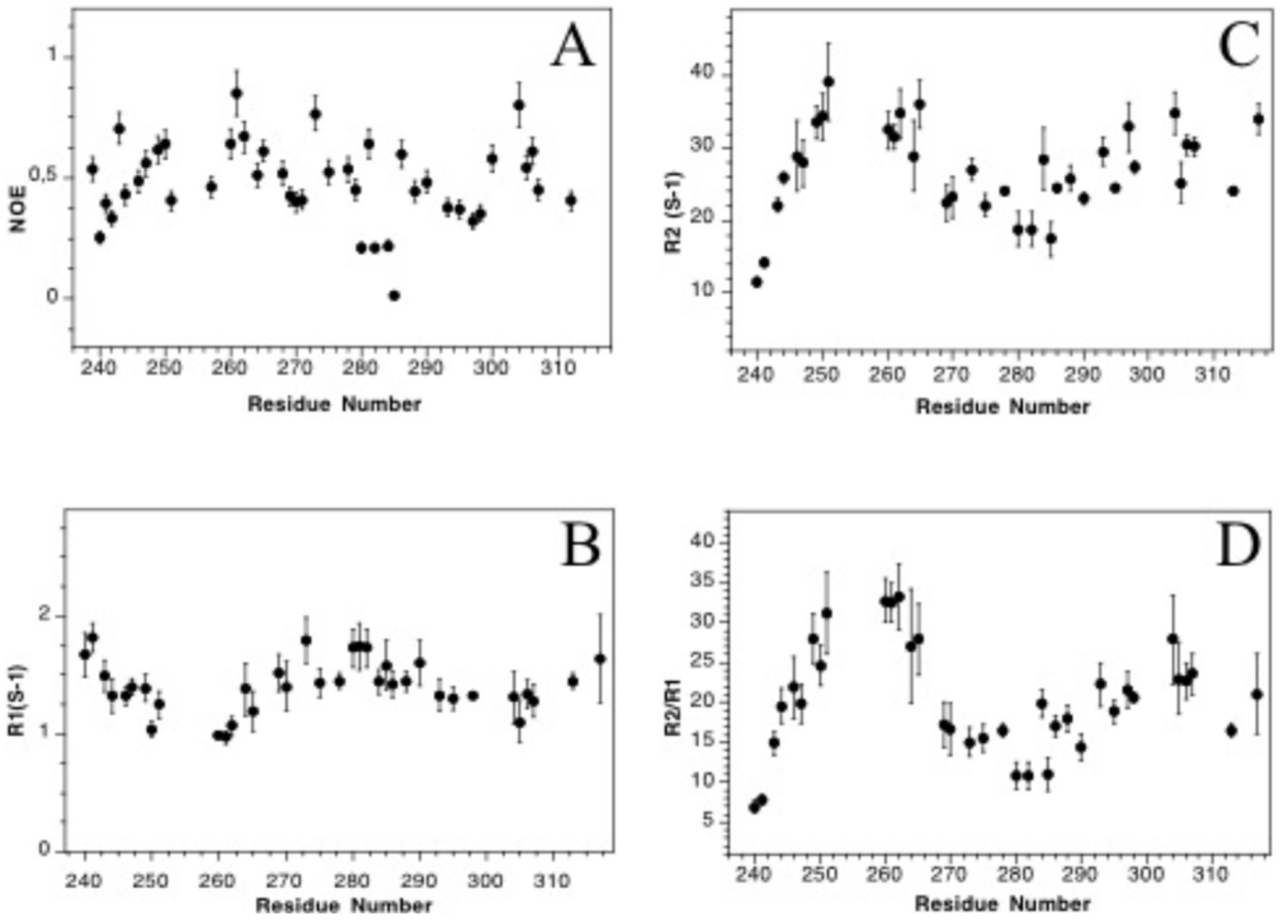
Backbone dynamics of HasS_CTD_. Steady state ^15^N-^1^H (A) longitudinal and transverse relaxation rates R1 (B) and R2 (C) were measured at 600 MHz and 20°C. R2/R1 ratios were calculated from B and C.

Additional information about HasS_CTD_ was obtained using ^15^N relaxation measurements, which are sensitive to overall and internal motions of the backbone amides. ^1^H-^15^N NOE values, sensitive to the fast internal dynamics, are shown in Figure 2A. Their average (0.5) is smaller than that obtained for a globular protein (around 0.8). Nevertheless the residues of HasS_CTD_ still display some restricted motions and are not highly flexible, as usually characterized by negative or very small positive values [29].

As shown in Figure 2D, the R2/R1 ratios of HasS_CTD_ are heterogeneous (ranging from 10 to 40) indicating a high degree of differential mobility throughout the protein sequence. There are at least three polypeptide segments with different dynamic properties similar to what was observed in the case of the partially folded A-state of ubiquitin [30]. Usually the R2/R1 ratios, depending on the overall dynamics of the proteins, are uniform across the structured parts of proteins tumbling in solution as unique globular units. In our case, the H2 helix displays the highest R2/R1 ratio suggesting that this region is the most restricted in motion.

### Interaction of HasS_CTD_ with the micelles of detergent

**Figure 3:**
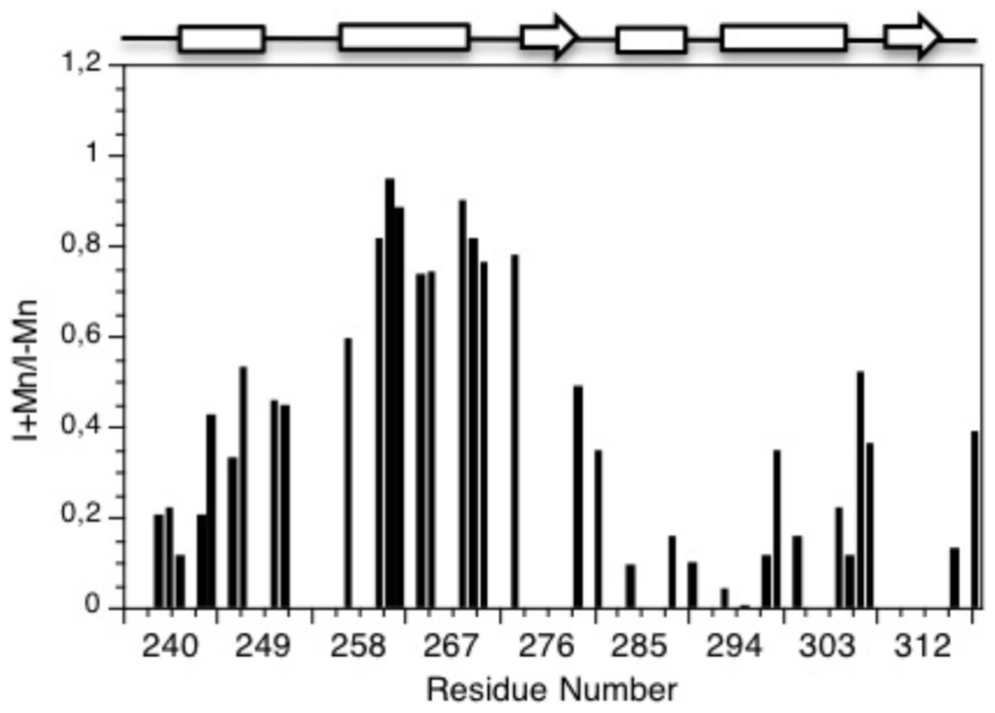
Ratio of the peak intensities of the two ^15^N-^1^H HSQC spectra (presence of Mn/absence of Mn). Overlapping peaks were not analysed. The secondary structure is displayed on the top of the figure with helices represented as rectangles and β strands as arrows.

As described above, after the refolding from the inclusion bodies, HasS_CTD_ remained stable in solution only in the presence of detergent micelles. We used the paramagnetic property of Mn^2+^ to determine by NMR which parts of the protein interact with the detergent. The unpaired electrons of Mn^2+^ induce broadening and thus disappearance of the peaks corresponding to the residues that are exposed to the solvent [31], [32]. In contrast, since Mn^2+^ cannot reach residues in the hydrophobic environment of the micelles, the NMR signals of these residues remain thus unaffected. As shown in Figure 3, all protons NH of HasS_CTD_ residues except those belonging to the H2 helix (T_257_QALAQLNRYR_267_) are highly affected by the presence of Mn^2+^. Therefore, only the H2 residues are not exposed to the solvent and thus seem to be in interaction with the molecules of detergent. This interaction between this helix and the molecules of detergent explains why this helix is the most restricted in motions.

### HasS_CTD_ affects *in vivo* expression of HasA and HasR

**Figure 4:**
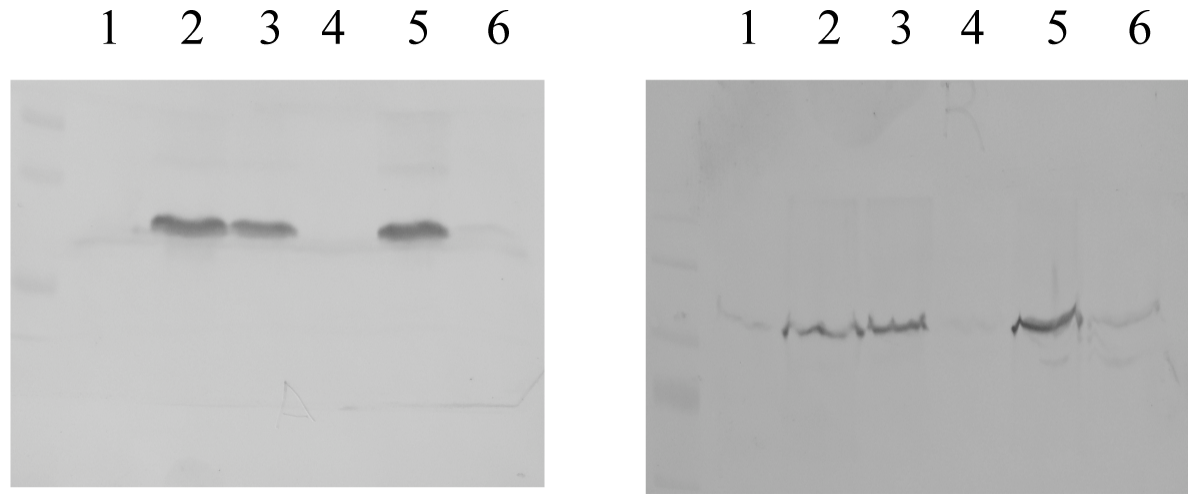
Influence of HasS_CTD_ expression on the regulation of the expression of HasR and HasA in *S. marcescens*. Immunodetection with anti-HasR (left panel) and anti-HasA (right panel) antibodies of whole cell extracts of *S.marcescens* SM365 either transformed with pBAD24 (control) or pBADHas_CTD_ for periplasmic expression of HasS_CTD_. Lane 1, SM365pBAD24 grown in presence of glucose and iron; lane 2, SM365pBAD24 grown in presence of glycerol, dipyridyl and heme; lane 3, SM365pBAD24 grown in presence of arabinose, dipyridyl and heme; lane 4, SM365pBADHasS_CTD_ grown in presence of glucose and iron; lane 5, SM365pBADHasS_CTD_ grown in presence of glycerol, dipyridyl and heme; lane 6, SM365pBADHasS_CTD_ grown in presence of arabinose, dipyridyl and heme.

HasS_CTD_ is predicted to reside in the periplasm. We have shown that this domain is partially unfolded and needs to be in the presence of detergent micelles mimicking membrane environment. To ascertain whether these structural features of HasS_CTD_ are compatible with its role in the signaling pathway, we have studied the effect of HasS_CTD_ on the regulation of the expression of HasR and HasA in *S. marcescens*. As a readout we detected the amount of both HasA and HasR in cultures from *S. marcescens* in various conditions, 90 minutes after the induction of the *has* operon. The Figure 4 shows the amounts of both HasA (19 kDa, right panel) and HasR (96 kDa, left panel) detected in whole cell cultures expressing or not HasS_CTD_, respectively shown at lane 4, 5 and 6 versus lane 1, 2 and 3. Different inducing conditions have been tested: iron rich (lanes 1 and 4) and iron starvation with low heme concentration (lanes 2,3,5 and 6). Neither HasR nor HasA are expressed under iron-rich conditions, whereas both are expressed in iron poor medium in the presence of heme. The expression of the HasS_CTD_ in the periplasm driven by plasmid pBAD24HasS_CTD_ in the presence of arabinose leads to a very strong reduction in both HasA and HasR expression, as compared to the control (lanes 6 *versus* 3). This result strongly suggests that HasS_CTD_ expression in the periplasm of S. *marcescens* interferes with the signaling cascade, most likely by trapping one of its essential components.

### Structure and dynamics of the periplasmic domain of HasR

**Figure 5:**
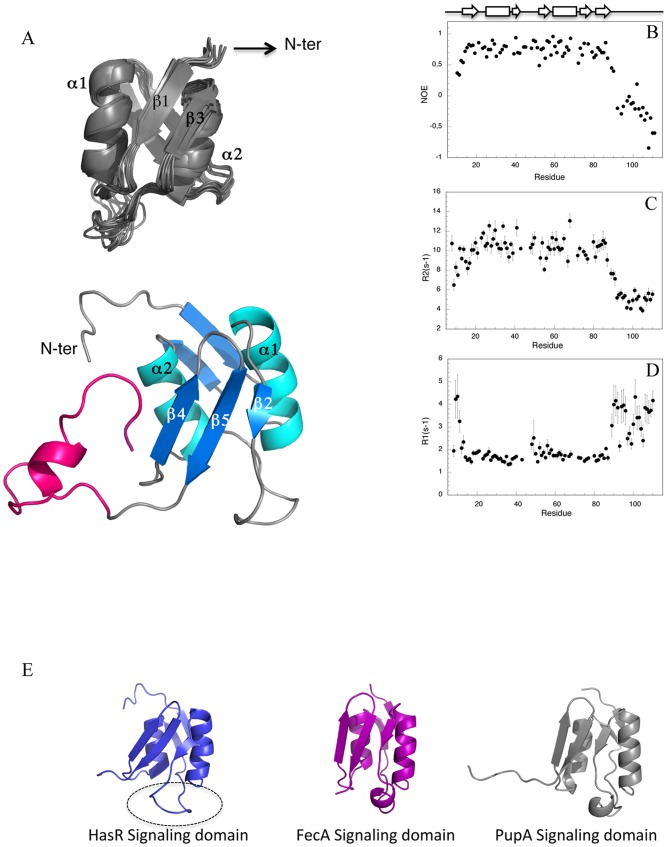
Structure and backbone dynamics of the periplasmic domain of HasR. (A) Overall structure of the HasR periplasmic domain. Top: cartoon diagram of the family of ten structures with lowest energy values (the flexible N- and C-termini are not presented). Bottom: (A) representative structure of the family (all residues are shown) with the linker containing the HasB/TonB box (magenta). The structure is rotated 180° around the Y axis. (B) Steady-state ^15^N-^1^H NOE (C) relaxation rates R2 and (D) R1 were measured at 600 MHz at 20°C. (E) Structural comparison of the signaling domain of HasR with its two structural homologous. The loop 35–47 is surrounded.

The periplasmic domain of HasR is believed to be the partner of HasS_CTD_ in the signaling pathway. To examine this interaction, we expressed and purified the periplasmic domain of HasR (signaling domain plus the region containing the TonB/HasB box) and solved its structure by NMR (Figure 5A). The details of the constraints and structural characteristics of the family of 20 conformers representing the solution structure of the periplasmic domain of HasR are summarized in Table S2 in File S1. The atomic coordinates of the ensemble of the 20 lowest energy conformers of the periplasmic domain of HasR have been deposited in RCSB Protein Data Bank *(* http://www.rcsb.org *)* under the accession code 2M5J.

The solution structure of the periplasmic domain of HasR exhibits two distinct regions (Figure 5A–D): a globular fold region corresponding to the signaling domain (residues 8–90) followed by an unstructured and flexible region comprising the 21 last residues. The dynamic properties of the protein backbone studied by measuring the ^15^N longitudinal (R1) and transverse (R2) relaxation rates together with steady-state ^15^N-^1^H NOE values also reveal a clear difference between these two regions. As can be seen in Figure 5B–D, the last 21 residues containing the TonB/HasB box display smaller values of R2 and higher values of R1 than their respective average for the whole protein. ^15^N-^1^H NOEs, very sensitive to the fast internal dynamics, have very small or negative values in this region indicating that it is flexible and unstructured.

The signaling domain (residues 8–90) shows a mixed α-β structural fold, with two α-helices sandwiched between two β-sheets, one anti-parallel, double-stranded (β1, β3), the other mixed, triple-stranded (β2, β4, β5) (Figure 5A).

The topology of the signaling domain of HasR is similar to that of other known signaling domains of TonB dependent transporters especially those of FpvA [33]–[35] and PupA [36], which are its closest structural neighbors found by Dali program [37]. However a slight difference can be observed in the long loop of 13 residues (40–52) connecting the β2 strand to the β3 strand. The corresponding region of other signaling domains contains a short helix, which is absent in the structure of HasR signaling domain. This loop displays particular dynamic properties and seems to undergo conformational exchange since its resonances are unusually weak. Indeed, some residues of this loop exhibit microsecond to millisecond time scale motions consistent with their high Rex values (>2.5 Hz) obtained from the analysis of relaxation parameters by Tensor program. Besides this loop, a few residues of the two α-helices are also affected by this type of motion as illustrated in Figure S3 in File S1.

### Interaction of HasS_CTD_ with the signaling domain of HasR

**Figure 6:**
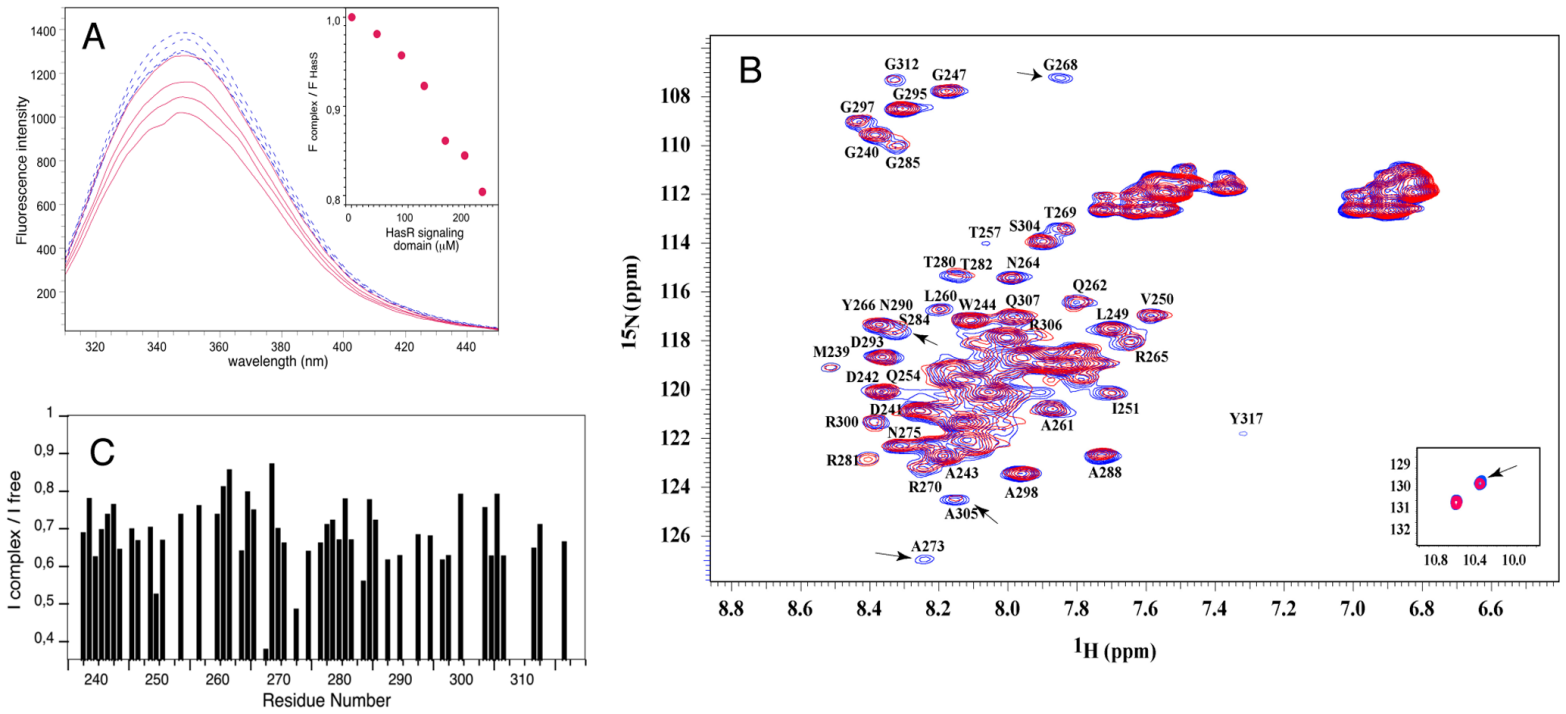
Interaction between HasS_CTD_ and the signaling domain probed by NMR and fluorescence. (A) Change of the fluorescence emission spectra of HasS_CTD_ upon titration with the signaling domain (solid red lines) and control spectra for HasS_CTD_ dilution with the buffer (dashed blue lines). Figure in the inset presents the ratio of the fluorescence intensity (at 350 nm) of HasS_CTD_ in complex and HasS_CTD_ alone, plotted versus the signaling domain concentration. (B) ^1^H-^15^N HSQC spectra of HasS_CTD_ alone (blue) and in the presence of unlabeled signaling domain (red). Some of the most affected residues are pointed out and resonances of tryptophan side chain are shown in the inset. The concentrations of HasS_CTD_ and the HasR signaling domain were 250 µM and 800 µM, respectively. (C) Ratio of the peak intensity of two ^15^N-^1^H HSQC spectra of ^15^N labeled HasS_CTD_ (in the presence and absence of the unlabeled signaling domain).

In order to obtain evidence of the interaction of HasS_CTD_ with the periplasmic domain of HasR during the signaling process, we have studied *in vitro* interaction of these two proteins. This protein-protein interaction was first monitored by fluorescence spectroscopy, taking advantage of the absence of tryptophan and tyrosine residues in the signaling domain of HasR. The intrinsic fluorescence spectra of HasS_CTD_ excited at 280 nm had a maximum at 350 nm and a shoulder with a maximum value around 335 nm showing that its two tryptophan residues are located in distinct environments, one of them being widely exposed to the solvent (Figure 6A). Addition of the signaling domain of HasR results in quenching of the HasS_CTD_ intrinsic fluorescence that can be associated with a change in the environment of the tryptophan side chains. This effect is proportional to the amount of the added signaling domain and therefore indicates the formation of a complex between HasS_CTD_ and the signaling domain of HasR (Figure 6A). It is not due to an unspecific protein-protein interaction, since a control experiment with Ribonuclease A does not show any significant change of the fluorescence signal of HasS_CTD_ (Figure S4 in File S1).

To further characterize this protein-protein interaction and identify the interface region(s), we used heteronuclear NMR. This technique allows to follow only the labeled protein, whilst its unlabeled partner remains invisible. The high sensitivity of the NMR signals relative to the size of the molecule and to the local environment of each observed nucleus enables the detection of molecular interactions even in the case of transient or low affinity complexes [38]. To do that, we compared the intensities and the chemical shifts of ^15^N-^1^H backbone peaks of the labeled protein before and after the addition of its unlabeled partner.

Figures 6B and C show the result of the analysis of the interaction between the HasR signaling domain and HasS_CTD_ when this latter is observed. Small shifts of certain resonances were observed. However, the major effect of the presence of the signaling domain on the HasS_CTD_ spectrum is the reduction of the overall intensity (about 35%). This change of intensity is triggered by addition of the signaling domain and is not due to a modification of HasS_CTD_ such as the aggregation of a population of proteins. Indeed, a sample of HasS_CTD_ does not show any reduction of intensity from one spectrum to another under the same conditions of concentration and buffer used for the interaction study.

The intensities and linewidths of NMR resonances can be affected by the molecular weight (lower intensity and line broadening with higher molecular size) and also by the exchange between the states (for example between free HasS_CTD_ and a complex with the signaling domain). Resonances of a few specific residues showed more reduction in intensity than its global value, strongly suggesting that these residues are in direct contact with the signaling domain. The following residues of HasS_CTD_ were the most affected upon binding to the signaling domain: G240, W244, V250, I251, G268, A273, N275, S284, A288, G297, A298, A305, Q307, G312.

Similar binding experiments and analysis were done with a sample containing labeled signaling domain and unlabeled HasS_CTD_. The residues of the signaling domain the most affected by the interaction were: L23, L28, R29, A31, A34, L53, V59, G62, I67, L76.

For this interaction study by NMR, either the signaling domain or the periplasmic domain of HasR (signaling domain plus the linker containing the HasB/TonB box) were used. The presence of the linker at the N-terminus of the signaling domain, did not modify either the interaction with HasS_CTD_ or the identified residues involved in this interaction.

## Discussion

On the basis of bioinformatic analysis of this family of antisigma factor and *in vivo* studies of the Fec system, we have defined three different C-terminal domains of HasS: an entire periplasmic domain, HasS_102_ corresponding to the region downstream from the putative transmembrane helix, HasS_166_ containing residues 166 to 317 starting after a stretch rich in hydrophobic residues and predicted as an additional putative transmembrane fragment, and HasS_CTD_ corresponding to the last 78 residues. HasS_CTD_ is similar to the shortest domain of FecR, the most studied antisigma factor of this family, defined as the domain of interaction with the transporter [12]. All our attempts to obtain soluble periplasmic domain of HasS, either HasS_102_, or HasS_166_, or HasS_CTD_ were unsuccessful despite using several expression vectors, strains and expression conditions. Our efforts were then focused on obtaining the shortest domain, HasS_CTD_ by a renaturation process from inclusion bodies. It was also elsewhere reported that the expression tests of FecR had led to its production in inclusion bodies [12].

Interestingly, after renaturation, HasS_CTD_ could only be kept in solution in the presence of the detergent micelles. Its structural characterization by NMR, CD and ultracentrifugation convincingly demonstrated that HasS_CTD_ is an elongated monomer in association with the detergent micelles. Using backbone NMR resonances, we have shown that HasS_CTD_ adopts secondary structures whose content matches those determined by Far-UV CD and that predicted *in silico,* suggesting that its “native-state” secondary structures may be present under our conditions. However, several lines of evidence suggest that its tertiary structure, if any, is not stable. The unusual broadening of the NMR resonances of HasS_CTD_, the behavior of the protein in thermal unfolding experiment and the absence of stabilized hydrogen bonds are clearly in favor of a “partially disordered” protein.

In order to ascertain whether HasS_CTD_ is in its active form, we wished to determine if it was able to fulfill its function. The expression of HasS_CTD_ in the periplasm of *Serratia marcescens* drastically decreases the amount of expression of both HasR and HasA upon induction of the *has* operon. This result strongly suggests that HasS_CTD_ is able to trap an essential component of the signaling cascade.

The potential partner of HasS_CTD_ is the periplasmic domain of HasR. We have expressed this domain and solved its 3D structure by NMR. It is composed of a globular part corresponding to the signaling domain followed by a flexible and disordered stretch of 21 residues. This fragment linking the signaling domain to the “plug-barrel” part of HasR is the main region of interaction with the energy transducer [10]. We have studied the interaction of HasS_CTD_ with the signaling domain of HasR by NMR and fluorescence. The presence of the linker containing the HasB/TonB box does not modify this interaction. We measured by NMR the variations of the intensities of the amide backbone resonances. This method is now increasingly used, especially in the case of low affinity or transient complexes [39]. In the study presented here, we were able to identify the interacting residues of both partners of the complex. However our attempts at using this information for docking HasS_CTD_ onto HasR signaling domain did not give conclusive results due to the fact that HasS, a 78 residues long protein is partially disordered. To our knowledge, there is currently no docking study involving a non-structured protein of this size [40], [41]. Nevertheless, the knowledge of the interacting residues allows to propose a mode of interaction between HasS_CTD_ and the signaling domain of HasR.

**Figure 7:**
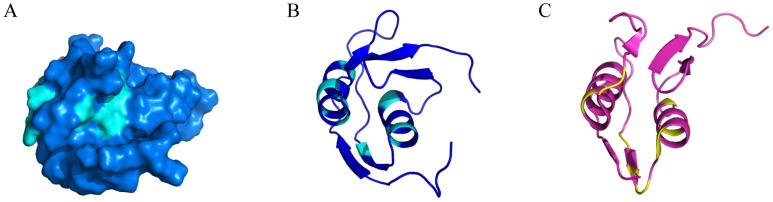
Residues of the signaling domains involved in the interaction with the antisigma factor. (A and B) The signaling domain of HasR (PDBid: 2M5J) is in blue and its residues involved in the interaction with HasS_CTD_ are in cyan. (C) The signaling domain of FecA (PDBid: 2D1U) is in pink [50] and its residues involved in the signaling process are in yellow.

**Figure 8:**
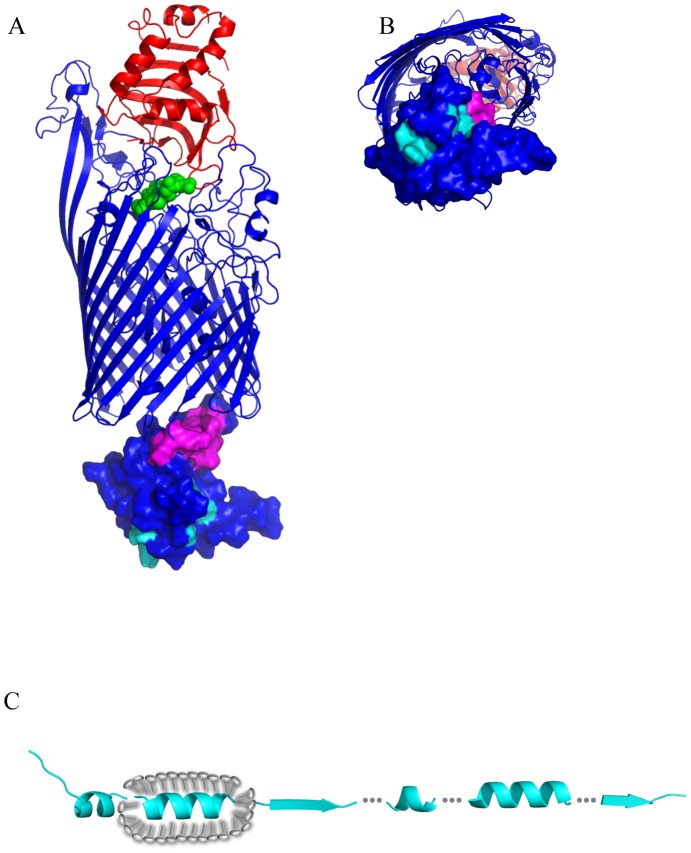
Structural representation of HasR receptor and HasS_CTD_. (A) The model of HasR receptor (in blue) containing the signaling domain was built with MODELLER using X-ray structure of the complex of HasR and HasA (PDBid 3CSL), the solution structure of the periplasmic domain of HasR described here and the X-ray structure of FpvA of *Pseudomonas aeruginosa* (PDBid 2O5P), the transporter of pyoverdine siderophore. HasA is colored in red and the residues of the signaling domain involved in the interaction with HasS and HasB are colored in cyan and magenta, respectively. Heme is in green (B) Bottom view of A. (C) Secondary structure representation of HasS_CTD_ (cyan) and the H2 helix association with the micelle of detergent (gray). Dashed lines represent the non structured parts. The figure is not drawn to scale.

On the signaling domain side, two regions are involved in the interaction with HasS (Figure 7A and B). One, located on the protein surface is highly accessible. The other is found in the groove between the two helices (Figure 7A and B). Interestingly, the residues of these helices exhibit microsecond to millisecond time scale motions in agreement with their high Rex values (Figure S5 in File S1). This type of motion was shown to be involved in molecular recognition process [42]. In a random mutagenesis study of the Fec system [43], the mutation of several residues of the signaling domain highly affected its function. The most affected mutation sites are presented in Figure 7C. Their localization corresponds to the regions of interaction we defined for our system. In addition, as shown in our model of the entire HasR, the interface regions have an optimal location for recognition by HasS (Figure 8A and B).

**Figure 9:**
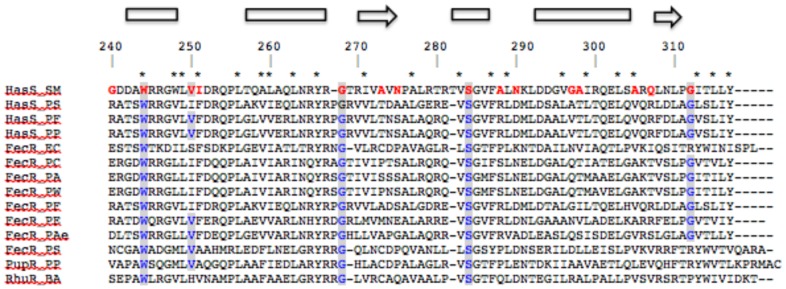
Sequence alignment of HasS and antisigma proteins. HasS_CTD_ residues involved in the interaction with the signaling domain are in red and their conserved equivalent in blue and highlighted. * represents conserved hydrophobic residues. Sequence numbering and secondary structure annotations are according to HasS_CTD_. HasS_SM: HasS of *Serratia marcescens*; HasS_PS: HasS of *Pseudomonas synxantha*; HasS_PF: HasS of *Pseudomonas. fluorescens*; HasS_PP: HasS of *Pseudomonas. protegens*, FecR_EC: FecR of *E. coli*; FecR_PC: FecR of *Pectobacterium carotovorum*; FecR_PA: FecR of *Pectobacterium atrosepticum*; FecR_PW: FecR of *Pectobacterium wasabiae*; FecR_PF: FecR of *Pseudomonas fluorescens*; FecR_PE: FecR like of *Pseudomonas entomophila*; FecR_Pae: FecR like of *Pseudomonas aeruginosa*; FecR_PS: FecR of *Pseudomonas synxantha*; PupR_PP: PupR of *Pseudomonas putida*; RhuR_BA: RhuR of *Bordetella avium*.

Several residues (W244, V250, G268, S284) of HasS interacting with the signaling domain are highly conserved in this family of antisigma proteins (Figure 9). The absence of conservation of the remaining residues, especially those of the C-terminal extremity, suggests that these residues may define, at least partly, the specificity of interaction and therefore signaling by the Has system.

We have shown by NMR that only one region of HasS_CTD_, the H2 helix (T_257_-QALAAQLNRYR_267_) interacts with the detergent micelles that could mimic the membrane environment. This behavior, consistent with the highly hydrophobic composition of this helix suggests that it could be in contact with membrane, most probably with the periplasmic face of the inner membrane. This potential interaction with the membrane could preserve the HasS periplasmic part from degradation by proteases. The hydrodynamic features obtained by analytical ultracentrifugation suggest that HasS_CTD_ has an elongated shape. The micelle of detergent around the H2 helix makes this part of the protein “more globular” than the regions outside of the micelles. Indeed, it has been shown that membrane proteins surrounded by molecules of detergent adopt some “cylindric/elliptic” form [44]. Based on the structural and hydrodynamic features of HasS_CTD_ we propose a model in which the H2 helix is embedded in the detergent micelle whereas the regions at its N- and C-terminal sides have an elongated shape and are solvent accessible (Figure 8C). The conservation of the secondary structure content and of hydrophobic residues in this family of antisigma factors suggests that the structural features of HasS_CTD_ may also exist for other proteins of this family (Figure 9).

The interaction of HasS_CTD_ with the membrane may keep it away from its partner, the signaling domain. Energized HasB/TonB is required for the regulatory function of the TonB-dependent transporters. Its role could be to trigger modifications of the structure and/or of positioning of either HasS or the signaling domain that would favor the interaction and the signaling process. One of the proposed effects of action of the energized TonB is pulling of the plug out of the barrel in a partially unfolded state, opening a path that allows the internalization of the nutrient through the transporter [45]. Here we have shown that the signaling domain of HasR is an independent structural domain which could stay globular when the plug becomes unfolded. The pulling mechanism of TonB/HasB might enable to displace the signaling domain and therefore to promote its interaction with HasS. Although there is no experimental evidence for this mechanism, some changes of positioning of signaling domain have been observed in the X-ray structures of FpvA (the transporter of pyoverdine siderophore) and proposed in the case of the ferric citrate transporter in a recent EPR study [33], [46].

We have revealed the existence of two regions of the signaling domain interacting with HasS_CTD_. Due to steric hindrance, these regions could be hardly accessible by a folded protein (Figure 7A). Therefore, we postulate that the partially disordered state of HasS_CTD_, its elongated form together with the interaction of its H2 helix with the membrane allow a “wrapping mode” of interaction between HasS_CTD_ and the signaling domain.

It is now accepted that intrinsically disordered proteins are involved in a wide range of cellular functions including transport, regulation of transcription and signaling [47], [48]. The number of examples of partially disordered proteins involved in various biological processes is increasing. Among them, a partially disordered antisigma factor has been reported [49]. The antisigma factor FlgM binds to and inhibits the activity of σ28 directing the transcription of proteins involved in the bacterial flagellum formation. FlgM is structurally disordered and when it interacts with its sigma factor, only one part of it becomes structured. Here, we show that HasS_CTD_ is partially disordered and that its conformation is compatible with its activity. Our first structural and molecular interaction study of a periplasmic domain of an antisigma factor and its partner provide essential elements to clarify the individual steps of the signaling. Further structural investigation of proteins involved in this process, either alone or in complex, would allow better understanding of the signaling pathway.

## Supporting Information

File S1 **Figure S1, Figure S2, Figure S3, Figure S4, Figure S5, Table S1, Table S2.**
(PDF)
