# Supplementary material for: Interaction of a Partially Disordered Antisigma Factor with Its Partner, the Signaling Domain of the TonB-Dependent Transporter HasR
Source: PLoS One. 2014 Apr 11;9(4):e89502. doi: 10.1371/journal.pone.0089502 (PMC3984077; doi:10.1371/journal.pone.0089502)
Supplement: File S1 — Figure S1, Figure S2, Figure S3, Figure S4, Figure S5, Table S1, Table S2. (PDF) [file pone.0089502.s001.pdf]

Inventory of Supporting Information

Figure S1, Figure S2, Figure S3, Figure S4, Figure S5, Table S1, Table S2

Figure S1. HasS domain organisation. TM represents transmembrane helix.

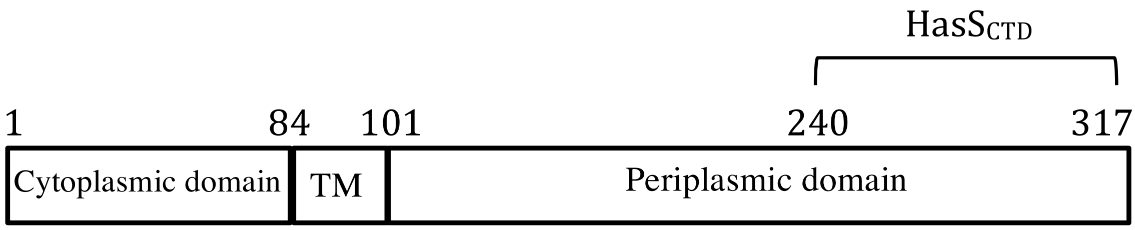

Figure S2. Purification of HasS$_{CTD}$. SDS PAGE of inclusion bodies (1) solubilized in 8 M Urea, elution profile and SDS PAGE of fractions (2) collected from the size exclusion column.

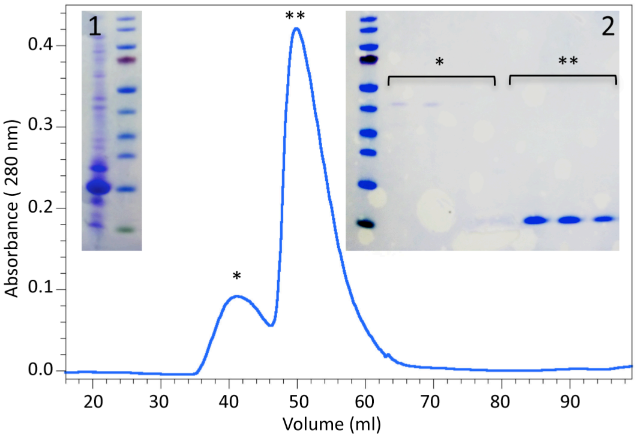

Figure S3. Summary of secondary Cα, Cβ and CO chemical shifts of Has_{CTD}.

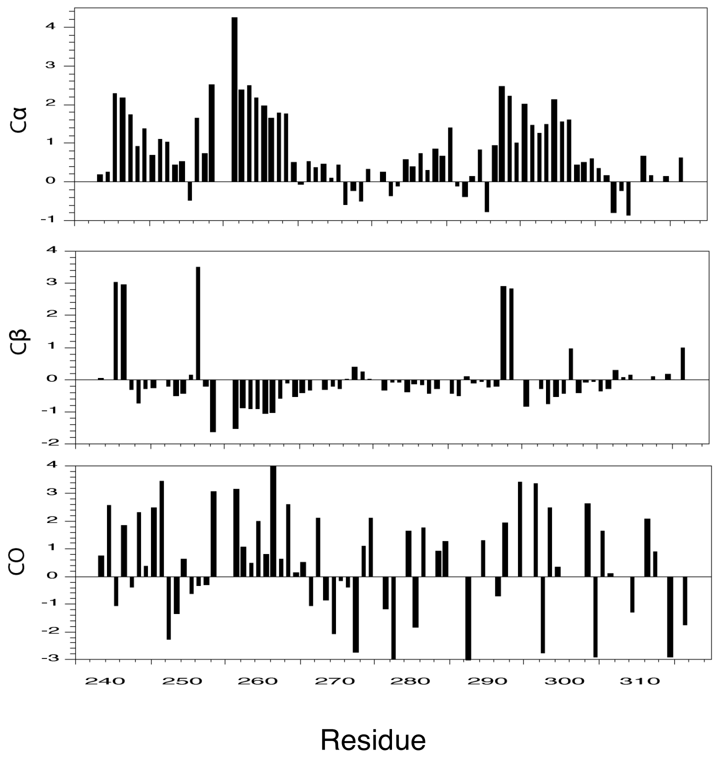

Figure S4. Variation of the fluorescence emission of $HasS_{CTD}$ upon titration with aliquots of a solution of HasR signaling domain (square) at 1 mM and ribunclease A (triangle) at 1.1 mM. The excitation and emission wavelengths were 297 and 350 nm, respectively.

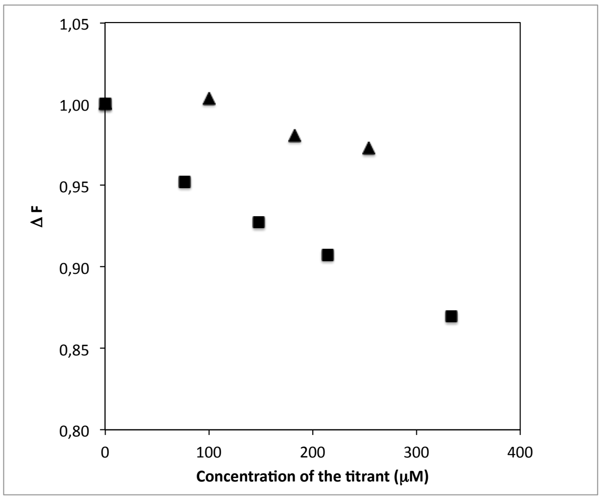

Figure S5. Residues of the signaling domain of HasR involved in the interaction with $HasS_{CTD}$ are in cyan and those affected by microsecond to millisecond time scale motions (Rex higher than 2.5 s^{-1}) are in red.

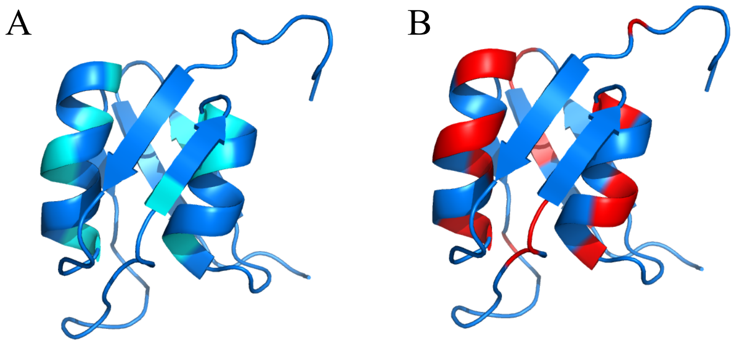

Table S1: $HasS_{CTD}$ chemical shift (in ppm)

		H	N	C	CA	CB
	Met	8.36	118.81	177.69	55.99	32.99
240	Gly	8.20	109.17	176.62	45.56	-
241	Asp	8.11	120.47	174.14	55.26	41.35
242	Asp	8.23	119.84	176.58	55.22	41.28
243	Ala	8.06	122.64	177.03	54.64	18.97
244	Trp	7.91	116.66	178.76	58.50	29.02
245	Arg	7.63	118.49	176.66	57.83	30.65
246	Arg	7.82	117.71	178.97	57.05	30.68
247	Gly	8.03	107.47	177.14	46.32	-
248	Trp	7.91	119.42	174.09	58.48	29.54
249	Leu	7.58	117.83	175.77	55.66	41.97
250	Val	7.48	117.03	176.68	62.85	32.40
251	Ile	7.64	119.90	175.65	61.14	39.05
252	Asp	8.01	121.98	174.83	54.54	41.83
253	Arg	7.98	117.88	175.70	57.21	30.72
254	Gln	8.29	119.86	176.68	56.70	27.90
257	Thr	8.00	112.80	178.03	66.26	68.48
258	Gln	7.93	120.50	176.95	58.56	28.64
259	Ala	7.91	121.87	178.15	55.22	18.37
260	Leu	8.02	116.20	179.27	57.53	41.55
261	Ala	7.76	120.54	178.55	54.77	18.22
262	Gln	7.70	115.91	180.17	57.78	28.51
263	Leu	7.85	118.69	177.91	57.27	41.87
264	Asn	7.84	115.32	177.76	54.99	38.98
265	Arg	7.55	117.76	176.23	57.01	30.40
266	Tyr	7.65	119.20	176.50	58.16	38.54
267	Arg	7.93	121.28	175.70	57.08	30.60
268	Gly	7.82	107.32	176.44	45.70	-
269	Thr	7.69	112.95	174.02	62.36	69.71
270	Arg	8.07	122.62	174.11	56.42	30.72
271	Ile	8.01	121.18	176.02	61.82	38.62
272	Val	7.73	119.80	175.45	61.80	32.83
273	Ala	7.98	125.70	174.90	52.33	19.64
274	Val	7.76	117.28	176.87	61.93	33.06
275	Asn	8.15	121.85	175.06	51.62	39.11
277	Ala	7.98	120.93	176.66	53.00	18.92
278	Leu	7.68	118.54	174.54	55.13	42.38
279	Arg	7.86	119.86	176.83	56.37	30.85
280	Thr	7.91	114.14	176.47	62.52	69.62
281	Arg	8.20	122.12	174.72	56.89	30.80
282	Thr	7.90	114.15	176.47	62.52	69.86
283	Val	7.95	119.81	176.83	62.98	32.38
284	Ser	8.08	116.79	175.90	59.43	63.78
285	Gly	8.14	109.55	175.14	45.91	-
286	Val	7.72	118.52	174.58	63.77	32.39
287	Phe	7.76	118.27	175.36	57.79	39.24
288	Ala	7.61	122.49	174.58	52.35	19.36
289	Leu	7.90	119.86	177.09	55.63	42.36
290	Asn	8.20	116.98	176.61	54.08	39.03

291	Lys	7.83	118.55	175.03	56.06	32.98
292	Leu	8.11	121.87	176.62	56.38	42.24
293	Asp	8.18	118.38	177.10	55.50	41.24
294	Asp	7.95	119.69	174.62	55.41	41.17
295	Gly	8.21	108.00	177.59	46.43	-
296	Val	7.96	118.68	174.54	64.59	32.00
297	Gly	8.29	108.87	177.12	46.59	-
298	Ala	7.85	122.99	175.07	53.89	18.98
299	Ile	7.78	117.61	178.97	63.08	38.15
300	Arg	8.16	121.03	176.66	58.53	30.39
301	Gln	8.02	118.86	177.54	57.70	29.10
302	Glu	8.01	119.86	177.13	57.65	29.84
303	Leu	7.95	119.14	177.32	55.87	42.06
304	Ser	7.77	113.69	177.04	59.04	63.97
305	Ala	8.02	124.22	174.89	53.48	19.20
306	Arg	7.89	117.41	178.00	56.82	30.57
307	Gln	7.86	116.92	176.11	56.23	29.24
308	Leu	7.91	120.43	175.29	54.69	42.75
309	Asn	8.02	119.15	177.54	52.82	39.17
310	Leu	7.91	121.80	173.97	52.86	42.60
312	Gly	8.18	107.05	176.23	45.88	-
313	Ile	7.69	118.54	177.66	61.77	39.01
315	Leu	7.82	121.88	174.36	55.59	42.63
317	Tyr	7.22	121.79	174.80	58.95	39.92

Table S2. Summary of the NMR constraints used for the structure calculation, the restraint violations and structural statistics for the ensemble of 20 best conformers of the HasR signaling domain.

NOE-derived constraints:	1141	*Energies* (kcal/mol)	
unambiguous:	943	E_{noe}	15.5 ± 3.5
intraresidue	420	E_{bond}	15.2 ± 0.9
sequential	235	E_{angle}	90.4 ± 6.4
medium range	80	E_{vdv}	-349.5 ± 19.2
long range	208	$E_{overall}$	-3524.0 ± 52.3
ambiguous:	198	*Mean pairwise RMSD* (Å) residues 8-82:	
intraresidue	102	Backbone atoms:	1.41 ± 0.39
sequential	45	All heavy atoms:	2.21 ± 0.40
medium range	11	*For the residues with regular 2ndary structure*	
long range	40	Backbone atoms:	0.81 ± 0.27
Hydrogen bonds:	24	All heavy atoms:	1.52 ± 0.25
DANGLE (φ,ψ):	176		
Total number of experimental. restraints	1341	*Ensemble Ramachandran plot* residues 8-82:	
		most favoured	83.7%
Residual distance constraint violations:		additional allowed	16.7%
>=0.5 Å	0.28 ±0.70	generously allowed	0.0%
>=0.3 Å	0.87 ±1.14	disallowed	0.0%
